# Effect of an organised screening program on socioeconomic inequalities in mammography practice, knowledge and attitudes

**DOI:** 10.1186/s12939-018-0811-3

**Published:** 2018-07-03

**Authors:** A. Relecom, B. Arzel, T. Perneger

**Affiliations:** 1 Division of Clinical Epidemiology, Geneva University Hospitals, rue Gabrielle-Perret-Gentil 4, 1211 Geneva, Switzerland; 2 Faculty of Medicine, University of Geneva, Geneva, Switzerland; 3 Oncology Unit, Geneva University Hospitals, rue Gabrielle-Perret-Gentil 4, 1211 Geneva, Switzerland; 4 Geneva Foundation for Breast Cancer Screening, Boulevard de la Cluse 43, 1205 Geneva, Switzerland

**Keywords:** Socioeconomic gradients, Mammography breast cancer screening, Organized screening program

## Abstract

**Background:**

Breast cancer stands as the leading cause of cancer related mortality in women worldwide. Mammography screening has the potential to improve prognosis by reducing stage at diagnosis. Socioeconomic inequalities in mammography cancer screening have been widely reported. The influence of organised programs on socioeconomic disparities regarding mammography screening is to date unclear. We aimed to investigate the impact of an organised regional screening program on socioeconomic inequalities in terms of the uptake, knowledge and attitudes towards mammography screening.

**Methods:**

Data were obtained from two cross-sectional surveys of women 50 to 69 years old conducted in 1998 and 2012, before and after the implementation of an organised breast cancer screening program in Geneva, Switzerland. Socioeconomic status was measured by monthly household income and education level. Logistic and linear regression multivariable models were used to investigate the evolution of socioeconomic gradients between 1998 and 2012 in terms of uptake, knowledge and attitudes towards mammography screening.

**Results:**

In 1998, before the implementation of an organised screening program, 44% of women from the lowest education category reported mammography practice conforming to recommendations versus 63% of the more educated participants. This socioeconomic gradient was no longer present in 2012 where reported mammography practice at guideline-recommended frequency were 83 and 82% in the lowest and highest education level categories respectively (change in education gradient over time, *p* = 0.018). The difference in mammography practice in agreement with recommendations between the lowest and the highest income category went from 27 percentage points in 1998 to 14 percentage points in 2012 (change in income gradient over time, *p* = 0.10). The socioeconomic gradient in negative attitudes towards mammography screening persisted in 2012 but was reduced compared to 1998. We did not observe a reduction in the socioeconomic disparities in knowledge regarding mammography screening over this period.

**Conclusions:**

This study suggests that mammography screening programs may lessen socioeconomic inequities in mammography practice. Such programs should feature adapted communication tools to reach women of lower socioeconomic status to attempt to further reduce socioeconomic gradients in mammography screening.

## Background

Breast cancer stands as the leading cause of cancer related deaths in women worldwide. With a breast cancer crude incidence rate of 153.5 per 100′000 women [1], Switzerland ranks amongst the most affected countries worldwide. Mammography screening offers the potential benefit of earlier stage at diagnosis allowing for an improved prognosis. A reduction in breast cancer mortality by mammography screening, whilst controversial in certain age categories, has been consistently observed in women 50 to 69 years old [2, 3].

Mammography screening may be available as part of an organized program which invites the defined target group to undertake the screening test at regular intervals. Such programs should operate with standardized equipment and procedures, allowing for quality control. Mammography screening carried out outside an organised program, either due to patient preference or to lack of availability of such a program, is defined as opportunistic screening [4]. WHO recommends organized population-based mammography screening programs for women of age 50 to 69 [5]. The benefit of such programs remains nonetheless debated by some, mainly due to concerns of overdiagnosis and potential harm caused by false positive tests [6].

Socioeconomic inequalities in breast cancer survival have been documented by a number of studies [7–9]. More advanced stage at diagnosis in the unprivileged contributes to increased breast cancer mortality in lower socioeconomic status groups [10, 11]. Features of organised mammography screening programs may allow them to contribute to lessen socioeconomic inequalities in breast cancer mortality by improving access to mammography in the more deprived.

An organised mammography screening program was implemented in the Geneva canton in Switzerland in 1999. Under this program, women aged 50 to 74 years old are systematically invited every 2 years to undertake a mammography screening test in a certified radiology unit. Mammography carried out as part of this program is not subject to the deductible of the health insurance and is covered free of charge for individuals receiving a subsidy for their health insurance. This limitation in ‘out of pocket’ payments may provide an incentive for mammography screening in the more deprived. This program also features mass media breast awareness campaigns addressing lack of knowledge and negative attitudes towards mammography practice, which are important barriers to screening attendance in lower socioeconomic groups [12, 13].

Our aim was to investigate whether the running of an organised screening program limiting ‘out of pocket’ payments and featuring breast cancer awareness campaigns was accompanied by a change in socioeconomic gradients in terms of uptake, knowledge of and negative attitudes towards mammography screening.

## Methods

In 1998, prior to the introduction of the regional organised program, self-completed questionnaires were sent to 1400 district households by an independent office with access to an exhaustive registry of residents of the Geneva canton of Switzerland. Households where women aged 40 to 79 were registered were selected and assigned a random number. The mailing list for the questionnaire was issued by selection of the first 1400 individuals from this list sorted by the randomly assigned number. This questionnaire was designed to investigate mammography practice and attitudes towards mammography screening in the Geneva population. A similar cross-sectional survey study was repeated in 2012 so as to evaluate the evolution in the previously mentioned outcomes in the 14 years since the launch of the screening program. The 2012 questionnaire was sent to 2000 women whose addresses were selected (using the same randomized selection process as outlined above for the 1998 questionnaire) this time from the list used by the program for their screening invitations [14, 15]. This is also an exhaustive list of legal residents of the canton from which all women in the age group targeted by the screening program can be identified. Only women 50 to 69 years old were included in this study. The questionnaires were structured in a similar fashion, collecting information on sociodemographic factors, past mammography practice, intention to screen and assessing attitudes towards mammography screening.

Mammography screening uptake was assessed by the proportion of participants who reported having had mammograms at the recommended frequency in the past 4 years. The questionnaires included 8 statements that tested knowledge of mammography screening. These items were scored on a 5-point Likert scale ranging from “I totally agree” to “I totally disagree”. To facilitate interpretation, the sum of correct answers was transformed into a mammography screening knowledge scale from 0 to 100. Negative attitudes towards mammography screening were measured by Rakowski’s 5-item scale of cons [14, 16], which has been used and validated in this setting [17]. Summary scores of cons were also repoted on a scale ranging from 0 to 100.

Reported household income and education level were used as measures of socioeconomic status (SES), both being validated SES proxies. Women were asked to report their monthly household income within five predefined income categories. Information regarding education level was collected as the total years of education, including primary and secondary school, university and apprenticeship years. For statistical analysis, education level was then converted to a categorical variable (≤ 10 years of education, 11–13 years, 14–16 years, and ≥ 17 years). A copy of the original questionnaires is available (in their original French language form) upon request by contacting the last author.

Socioeconomic gradients in mammography practice were assessed separately for 1998 and 2012 using cross-tabulation and multivariable logistic regression models. In these models, adherence to mammography screening recommendations was set as the dependent variable and the socioeconomic marker as an independent variable, adjusting for age and marital status. Separate models for household income and education level as SES markers were used. The analysis was then completed by multivariable models setting as independent variables the socioeconomic position marker (household income or education level), the survey year (coding the year 1998 as “0” and year 2012 as “1”) and the interaction term of these two variables. The *p* value of this interaction term assessed the statistical significance of the change in the effect of the SES on mammography uptake over the running time of the program.

The socioeconomic gradients in knowledge and negative attitudes towards mammography in 1998 and 2012 were analysed using the same methodology, this time using linear regression models for these two continuous variables. Mammography screening knowledge scores and negative attitudes towards mammography scores were set as the dependent variables of the models and the independent variables used were the same as those described above for the “adherence to mammography screening recommendations” models. We used statistical software Stata 13.0 for all the analyses. *P* values of less than 0.05 were considered as statistically significant.

## Results

The survey response rate was 71.8% in 1998 which corresponds to 958 returned questionnaires out of the 1334 that were mailed. After further exclusion of 431 participants younger than age 50 or older than age 70, 521 surveys from 1998 remained for analysis. Of 1916 eligible women in 2012, 1083 responded (56.5%) and were included in the analysis. Education level was reported by 91.1% of participants and monthly household income by 85.9%. Age distribution was similar in both surveys.

The proportion of women who reported a high household income (>8000CHF/month) was significantly higher in 2012 than in 1998 and so was the proportion of women who reported a high level of education.

Participant characteristics by survey year are shown in Table 1. Nearly all women (97.3%) indicated ever having had a screening mammogram in 2012 compared to 86.3% in 1998 (*P* < 0.001). Similarly, 82.2% of women contacted in 2012 reported having had 2 screening mammograms or more over the past 4 years compared to 52.6% of women contacted in 1998 (*P* < 0.001). Mean knowledge score was 6.1 points higher (*P* < 0.001), and mean score of negative attitude towards mammography 10.2 points lower (*P* < 0.001) in 2012 than in 1998.

**Table 1:** Participants characteristics, practice, knowledge and attitude towards screening mammography, according to survey year

Participant’s characteristics	1998 (*N* = 521)	2012 (*N* = 1083)	*p* value
Age group (years)	N(%)	N(%)	
50–54	165 (32.9)	310 (29.2)	*p* = 0.1
55–59	131 (26.1)	253 (23.8)	
60–64	94 (18.8)	245 (23.1)	
65–69	111 (22.2)	254 (23.9)	
Monthly household income (CHF)			
< 2000	38 (9.2)	67 (7.0)	*p* = 0.006
2000–3999	93 (22.4)	188 (19.5)	
4000–5999	109 (26.3)	203 (21.1)	
6000–7999	79 (19.0)	196 (20.4)	
≥ 8000	96 (23.1)	309 (32.1)	
Education (years)			
0–10	150 (31.3)	195 (19.8)	*p* < 0.001
11 → 13	142 (29.6)	259 (26.3)	
14 → 16	107 (22.3)	263 (26.8)	
≥ 17	80 (16.7)	266 (27.1)	
Smoker	82 (16.0)	200 (19.3)	*p* < 0.001
In a relationship	336 (66.4)	695 (66.7)	*p* = 0.2
Study outcomes			
Ever had a screening mammogram	449 (86.3)	1041 (97.3)	*p* < 0.001
At least 2 screening mammograms over the past 4 years	254 (52.6)	860 (82.2)	*p* < 0.001
	Mean score/100 (SD)	Mean score /100 (SD)	
Screening mammography knowledge	67.7 (17.3)	73.8 (14.6)	*p* < 0.0001
Rakowski’s scale of cons	24.0 (23.0)	13.8 (17.7)	p < 0.0001

**Fig. 1:**
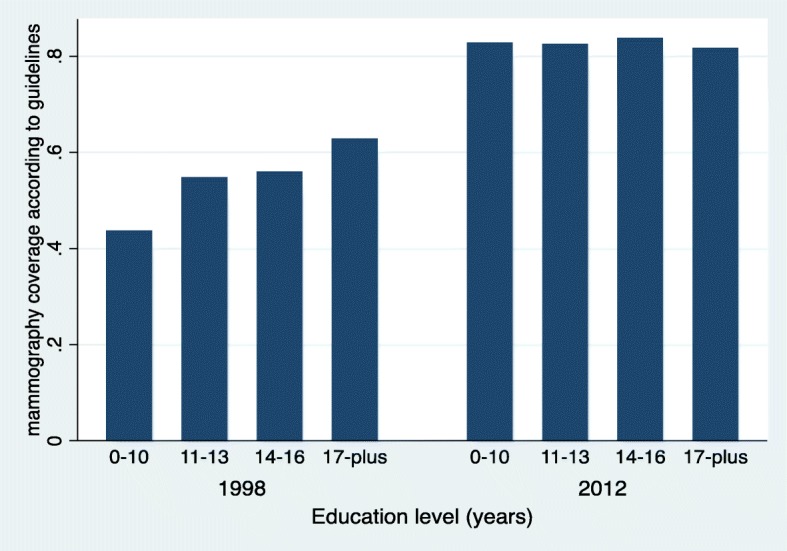
Reported screening mammography practice according to education category in 1998 and 2012. This figure shows a gradient in mammography uptake according to education category in 1998, which is no longer observed in 2012

**Fig. 2:**
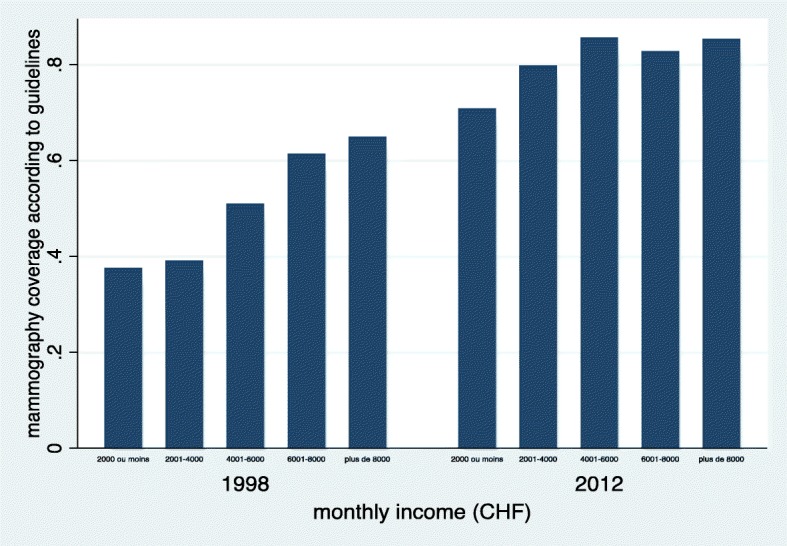
Reported screening mammography practice according to reported monthly household income category in 1998 and 2012. This figure shows a strong gradient in mammography uptake according to monthly income in 1998. This gradient is comparatively lessened in 2012

Socioeconomic gradients in the proportion of women who had 2 mammograms or more in the past 4 years in 1998 and 2012 are reported in Table 2 and illustrated graphically in Figs. 1 and 2. The strong.

**Table 2:** Knowledge and negative attitudes towards mammography screening according to household income and education level in 1998 and 2012

	Mammography practice according to guidelines (%)		Mammography screening knowledge (mean score/100)		Negative attitudes towards mammography screening (mean score/100)	
Education (years)	1998	2012	1998	2012	1998	2012
< 10	43.7	82.8	65.1	71.6	30.0	17.2
11–13	54.8	82.5	70.2	74.6	22.4	13.1
14–16	56.0	83.7	66.8	74.5	21.6	11.6
≥ 17	62.8	81.7	70.8	76.2	17.8	12.3
p value for trend	p = 0.012	p = 0.74	*p* = 0.028	*p* = 0.001	*p* = 0.002	*p* = 0.004
Monthly household income (CHF)						
< 2000	37.5	70.8	64.8	70.7	32.3	21.1
2000–3999	39.0	79.8	63.2	69.7	32.6	20.1
4000–5999	51.0	85.6	68.3	72.6	22.1	12.5
6000–7999	61.3	82.7	72.5	75.1	16.4	11.1
≥ 8000	64.9	85.3	70.3	78.1	16.9	9.9
p value for trend	p < 0.001	p = 0.024	*p* = 0.009	p < 0.001	p < 0.001	p < 0.001

positive linear association between education level and adherence to recommendation in mammography practice in 1998 was absent in 2012 (Fig. 1). The association between mammography screening practice and reported monthly household income was also stronger in 1998 than in 2012 (Fig. 2). In logistic regression (Table 3), the odds ratio for the increase in adherence to mammography practice recommendations from one “education level” category to the next went from 1.25 (*p* = 0.012) in 1998 to 1.03 (*p* = 0.74) in 2012. The reduction in the influence of education level on mammography uptake over the 14 years of running of the screening program was statistically significant (*p* = 0.018). Using household income as the independent variable, the odds ratio went from 1.36 (*p* < 0.001) in 1998 to 1.17 (*p* = 0.024) in 2012. The change in the effect of income on mammography uptake between 1998 and 2012 was not statistically significant (*p* value for interaction term = 0.10).

**Table 3:** Socioeconomic gradients in practice, knowledge and negative attitudes towards mammography screening

		Socioeconomic gradients in outcomes^a^		*p* value for change between 1998 and 2012 (interaction term)
Outcome	Socioeconomic marker	1998	2012	
		OR between adjacent categories of SE marker		
Reporting at least 2 screening mammograms over the past 4 years	Income^b^	1.36 (*p* < 0.001)	1.17(*p* = 0.024)	0.10
	Education^c^	1.25 (*p* = 0.012)	1.03 (p = 0.74)	0.018
		Difference in mean scores between adjacent categories of SE marker		
Knowledge regarding mammography screening	Income	2.24 (p = 0.009)	2.85 (p < 0.001)	0.63
	Education	1.73 (*p* = 0.028)	1.39 (*p* = 0.001)	0.34
Score of negative attitude towards mammography screening	Income	−3.89 (p = < 0.001)	−3.05 (p < 0.001)	0.041
	Education	−2.96 (*p* = 0.002)	−1.44 (*p* = 0.004)	0.025

^a^Reported values correspond to the regression coefficients of the SES variable issued from models detailed in the text

^b^income refers to reported household income used as a categorical variable in the model, with predefined income categories as detailed in the method section

^c^education refers to the reported number of years of education of participants, used as a categorical variable in the model using predefined education categories as detailed in the method section

Mammography screening knowledge was positively associated with household income and education level in both 1998 and 2012 (Table 2). The change in socioeconomic gradients regarding mammography screening knowledge between 1998 and 2012 (Table 3) was small and statistically non-significant, whether using education level or household income as socioeconomic level markers (p value for interaction term = 0.92 and 0.73 respectively).

We observed a significant inverse association between education level and negative attitudes toward mammography screening in both 1998 and 2012 (Table 2). The difference in mean score of negative attitudes towards mammography from one education level category to the next went from 2.96 in 1998 to 1.44 in 2012. The change in education level gradient in negative attitudes towards mammography between 1998 and 2012 was statistically significant (*p* = 0.025). A similar trend was observed using household income as a socioeconomic marker.

## Discussion

This repeated cross-sectional study suggests some improvement in socioeconomic inequities regarding mammography uptake over the running time of an organised screening program in the Geneva canton. The strong socioeconomic gradient in mammography screening adherence observed in 1998 was no longer present in 2012 if one considered education level as the socioeconomic marker. Although a similar pattern was observed with reported household income as a socioeconomic marker, the change in gradient was in this case only partial and was not statistically significant; inequities in mammography uptake according to household income persisted in 2012.

Previously published studies tend to support the hypothesis that organised screening programs have a corrective effect on socioeconomic gradients in screening mammography practice observed in the opportunistic setting. Palència et al. [18] reported an absence of socioeconomic inequalities in breast and cervical cancer screening in European countries running organised national screening programs whereas disparities persisted in countries without such programs. The more pronounced socioeconomic inequities in PAP smear screening practice as compared to mammography screening in settings where an organized screening program was running for breast cancer but not for cervical cancer also supports the capacity of organized programs to reduce socioeconomic gradients [19]. Socioeconomic inequalities in terms of breast cancer survival were also reduced after the implantation of an organised screening program in Florence, Italy [20]. However, others found no reduction in the socioeconomic gradient in mammography uptake before and 3 years after the implementation of an organised breast screening program in Belgium [21]. It has been argued that new public health measures take time to reduce health inequities and can even lead to a temporary widening of socioeconomic gaps [22] and the short time interval between the two evaluations in this study may account for these negative results.

This study suggests that knowledge regarding mammography screening has on the whole increased and negative attitudes lessened since the introduction of the organised program. Mass media breast screening awareness campaigns and the mailing of information leaflets regarding breast cancer screening to all women invited to have a mammogram may have contributed to this result. Our results however show that the substantial inequities in mammography screening knowledge observed in 1998 persisted in 2012. This was also the case for negative attitudes towards mammography, although the change in the effect of the SES on negative attitudes regarding mammography significantly declined over the 14 years of running of the program. This suggests that the features of the organized screening program addressing mammography screening knowledge and negative attitudes towards mammography described above have a limited reach and impact on women of lower SES. This leaves an opportunity for improvement and should stress the importance of adapting the program’s communication strategy in this regard. Communication campaigns focused on primary care physicians may be an important aspect of organised programs in this regard, knowing the impact physician recommendations have on mammography practice [14, 23, 24].

This study had several limitations. Our study was conducted in a defined area of Switzerland. We cannot ascertain that our findings can be extrapolated to other settings running organised screening programs with different features. The survey response rate was lower in 2012 than in 1998. Since information regarding non-responders was not available, we could not quantify the extent to which selection bias affected study results. Another limitation is the repeated cross-sectional study design. This implies that other factors than the introduction of the screening programme may have influenced the observed change in women’s behavior between 1998 and 2012. Also, even though we believe that the population has remained stable, unobserved confounders may have contributed to the observed changes. Monthly income and education level were also self-reported with no external validation. Finally, we also acknowledge that mammography screening uptake was assessed based on self-reporting. Whilst self reporting in survey data has been shown to overestimate cancer screening utilization, socioeconomic disparities tend to be masked by self-report [25]. This bias would be conservative in our study findings.

## Conclusions

This study suggests that mammography screening programs may lessen socioeconomic inequities in mammography practice. Such programs should feature adapted communication tools to reach women of lower socioeconomic status to in an attempt to further reduce socioeconomic gradients in mammography screening.

## Abbreviations

SES: socioeconomic status
